# Highly active nano-sized iridium catalysts: synthesis and *operando* spectroscopy in a proton exchange membrane electrolyzer†
†Electronic supplementary information (ESI) available. See DOI: 10.1039/c8sc00555a


**DOI:** 10.1039/c8sc00555a

**Published:** 2018-02-20

**Authors:** P. Lettenmeier, J. Majchel, L. Wang, V. A. Saveleva, S. Zafeiratos, E. R. Savinova, J.-J. Gallet, F. Bournel, A. S. Gago, K. A. Friedrich

**Affiliations:** a Institute of Engineering Thermodynamics , German Aerospace Center (DLR) , Pfaffenwaldring 38-40 , Stuttgart , 70569 , Germany . Email: aldo.gago@dlr.de; b Institut de Chimie et Procédés pour l'Energie, l'Environnement et la Santé , UMR 7515 , du CNRS-Université de Strasbourg , 25 Rue Becquerel , 67087 Strasbourg , France; c Laboratoire de Chimie Physique-Matière et Rayonnement , Sorbonne Université , UPMC Univ Paris 06 , CNRS , 4 place Jussieu , 75005 Paris , France; d Synchrotron-Soleil , L'orme des Merisiers , Saint Aubin , BP48 91192 Gif-sur-Yvette Cedex , France; e Institute of Energy Storage , University of Stuttgart , Keplerstraße 7 , Stuttgart 70174 , Germany

## Abstract

A stable and cost effective oxygen evolution reaction (OER) catalyst is crucial for the large-scale market penetration of proton exchange membrane (PEM) water electrolyzers. We show that the synthesis of iridium nanoparticles in either low purity ethanol or water, or in the absence of a surfactant, is detrimental to the electrocatalytic properties of the materials. Adding NaBH_4_ in excess improves the purity of the catalyst enhancing the OER activity up to 100 A g_Ir_^–1^ at 1.51 V *vs.* RHE, the highest value reported so far for high purity Ir nanoparticles. The measured OER activity correlates with the capacitive current rather than with the charge corresponding to the Ir^III^/Ir^IV^ oxidation peak. *Operando* near-ambient pressure X-ray photoelectron spectroscopy (NAP-XPS) on membrane electrode assemblies (MEAs) with the synthesized catalysts reveals a metallic core surrounded by a thin layer of Ir^III/IV^ oxides/hydroxides. Oxidation of Ir^III^ leaves behind a porous ultrathin layer of Ir^IV^ oxides/hydroxides, which dominate the surface during the OER, while Ir^V^ was not detected.

## Abstract 2

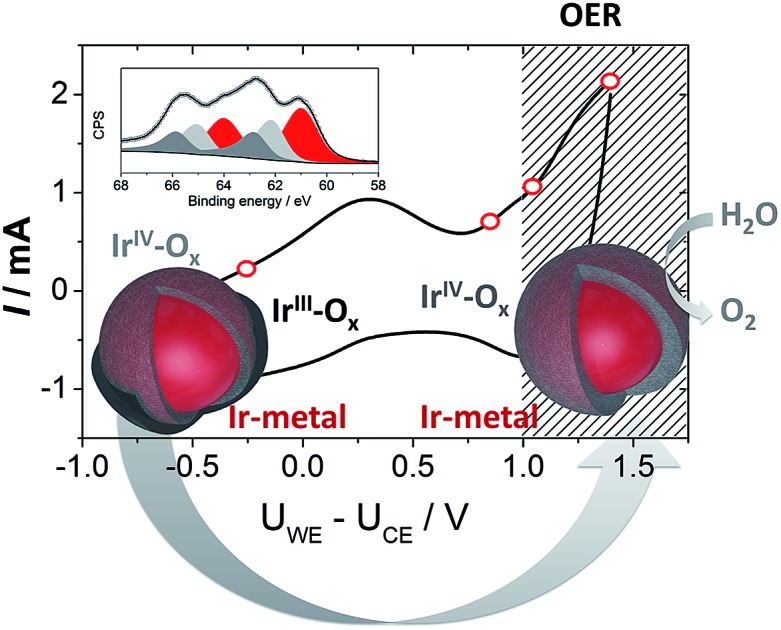
Ultra-high purity nano-sized iridium enclosed in a monolayer of Ir^III^/Ir^IV^ oxides/hydroxides leads to an enhancement in OER activity.

## Introduction

Recently published calculations by Rogelj *et al.*^1^ revealed alarming numbers regarding the climate change and the urgency for efficient energy systems. An early transition to net zero carbon emissions worldwide is required, calculated to be achieved between 2045 and 2060, to achieve the global warming limitation to below 1.5 °C, which is the goal of the Paris agreement.^2^ This agreement has been ratified by 146 parties to the convention and has thus officially entered into force.^3^ The timeframe is closing rapidly and the effort in substituting hydrocarbons by sustainable energy needs to be increased. Photovoltaic, wind and water turbine energies are currently the most promising paths to reduce the greenhouse gas emissions at the energy sector.

An additional pressing question is the transition of those sustainable energy sources into other highly energy-intensive sectors such as industry and transport. Therefore, hydrogen produced by electrolysis is a promising solution for storing renewable energy and can be used for producing synthetic fuels.^4^,^5^ Proton exchange membrane (PEM) water electrolysis is particularly interesting for such purposes, due to the high dynamic operation and the ability for partial- and overload. These are outperforming properties in use for grid services. Currently, PEM electrolyzer systems have power capacities up to 1.5 MW and are installed in container solutions.^6^ PEM electrolyzer systems have power capacities up to 1.5 MW, with efficiencies above 70% and are installed in container solutions.^6^ In 2003, the estimated worldwide production of hydrogen was approx. 500 bn Nm^3^ a^–1^.^7^ The major part of this production is based on hydrocarbons such as steam methane reforming or coal gasification and yet, PEM electrolyzer facilities are far away from meeting the hydrogen demands of industrialized countries.

As an example, 5% of the annual hydrogen production in Germany requires the installation of about 1 GW of PEM electrolyzers.^8^ The manufacture of 1 GW PEM electrolyzers would require approx. 0.6 t of iridium (taking into account the standard loading of the oxygen catalyst) which is about 10% of the annual mining/production of the precious metal.^9^,^10^

In terms of availability, the issue of using high loadings of Ir in membrane electrode assemblies (MEAs) is one of the most important research topics in PEM electrolysis. The target is to increase the electrocatalytic activity by the use of supporting materials,† 11–16 changing the crystalline structure,^17^–^19^ optimizing the electrochemical active surface area^20^–^23^ and to understand the degradation mechanism for such materials^24^,^25^ to decrease the required amount of Ir in highly efficient PEM electrolyzer systems.

Recent publications show a clear trend from stoichiometric rutile-type IrO_2_ catalysts towards materials with metallic character, IrO_*x*_–Ir. Without subsequent thermal treatment, electrochemically oxidized Ir-based catalysts show several orders of magnitude higher oxygen evolution reaction (OER) activity compared to the thermally oxidized counterparts. In this context, the group of Strasser produced highly active nanodendrites^13^ as well as IrNiO_*x*_ core–shell catalysts^26^ with superior activity and reported a decreasing performance with increasing the temperature of heat treatment. Moreover, Pi *et al.* reported Ir 3D-nanostructures with excellent activity in the alkaline and acidic media.^27^

Besides the OER activity, the catalyst cost and the complexity of the synthesis have a significant impact on the choice of the catalyst material of future electrolyzers. Based on previous published work,^22^,^28^ the following publication presents a cost-efficient synthesis route for highly active Ir-nanoparticles and discusses the influence of the quality and costs of the participating chemicals. Furthermore, we discuss various approaches to evaluate the electrocatalytic activity of the catalysts and use *operando* as well as *ex situ* characterization methods to unveil their composition and structure and explain their enhanced OER activity.

## Experimental

### Catalyst synthesis

**Fig. 1:**
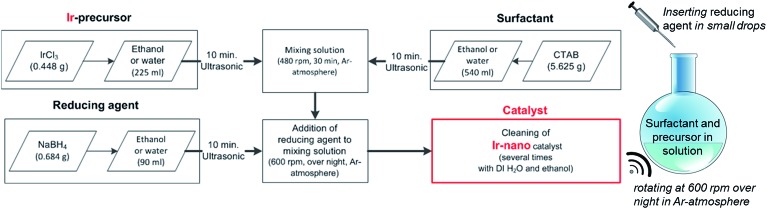
Scheme of the chemical synthesis procedure of Ir nanoparticles.

The herein presented catalysts are synthesized *via* a wet-chemical method shown in Fig. 1. To evaluate the influence of the solvent's purity, different grades are used for synthesis. Table 1 lists each investigated configuration. For producing iridium nanoparticles 5.625 g cetyltrimethylammonium bromide (CTAB) and 0.448 g iridium(iii) chloride (IrCl_3_) are dissolved in 540 ml and 225 ml ethanol (EtOH), respectively. After applying ultrasonication for 10 min each, the two solutions are mixed together using a magnetic stirrer (rotation speed at 480 rpm, 30 min). Meanwhile another solution of 0.684 g sodium borohydride (NaBH_4_) as the reducing agent and 90 ml ethanol is prepared with the assistance of an ultrasonication bath for 10 min. The latter solution is added at 2–3 ml min^–1^ to the former which is additionally purged with argon and now mixed at 600 rpm. This causes an obvious reduction of IrCl_3_ within an hour coloring the solution from greenish to blackish. The rotation speed is maintained for 12 hours to ensure an entire reduction.

**Table 1:** Precursors for the synthesis of Ir nanoparticles (Ir-nano)

Catalyst name	Solvent	Reductant	Ir precursor
Ir-nano 99.8	≥99.8% pure EtOH^*a*^	1× NaBH_4_	IrCl_3_^*b*^
Ir-nano 99.8-P	≥99.8% pure EtOH	5× NaBH_4_	IrCl_3_
Ir-nano 99.5	≥99.5% pure EtOH^*c*^	1× NaBH_4_	IrCl_3_
Ir-nano 99.5\CTAB	≥99.5% pure EtOH, without CTAB^*d*^	1× NaBH_4_	IrCl_3_
Ir-nano 91.5	≥91.5% pure EtOH^*e*^	2× NaBH_4_	IrCl_3_
Ir-nano H_2_O	DI water H_2_O^*f*^	2× NaBH_4_	IrCl_3_

^*a*^Ethanol absolute, water free (VWR Chemicals, product-no. 83672), purity ≥99.8%. Reference synthesis: 0.448 g IrCl_3_/5.265 g CTAB/0.684 g NaBH_4_/855 ml EtOH.

^*b*^Iridium(iii)-chloride, dried, min. 62% Ir (Alfa Aesar, product-no. 12158).

^*c*^Ethanol absolute (VWR Chemicals, product-no. 20816), purity ≥99.5%.

^*d*^Cetyltrimethyl ammonium bromide (VWR Chemicals, product-no. 22610.132).

^*e*^Ethanol 99% (VWR Chemicals, product-no. 84835), purity ≥91.5%.

^*f*^Water (VWR Chemicals, product-no. 90200), conductivity ≤1.1 μS cm^–1^.

Afterwards, the as-synthesized particles are separated from the liquid phase by a centrifuge (4 min at 7600 rpm). For removing contaminations the powder is washed four times with ethanol and four times with deionized water as well. Finally, the wet powder is dried in a furnace at 40 °C under an air atmosphere. Deviating from the above mentioned synthesis route, a five-fold amount of NaBH_4_ was used for Ir-nano 99.8-P. Furthermore, high purity EtOH – in the case of Ir-nano 99.8 and 99.8-P – was only exposed to argon during the wet-chemical synthesis. For that all flasks were purged with argon before being sealed. In order to investigate the influence of the EtOH quality, different samples with different purities of the solvent were prepared and characterized afterwards. Table 1 summarizes the chemical precursors for the synthesis of the catalysts. The influence of the surfactant CTAB as well as the use of water as a solvent was investigated too.

### Physical characterization

Scanning electron microscopy images were produced by using a Zeiss ULTRA plus with charge compensation in order to get information about the morphology of the catalysts. Detection of secondary electrons (SEs) and backscattered electrons (BSEs) with an accelerating voltage of 1 kV allows distinguishing different materials. Integrated energy-dispersive X-ray spectroscopy (EDX) was used for elemental analysis of the measured powders. At least eight measurements were recorded for each material. Samples for transmission electron microscopy (TEM) are prepared by dispersing the catalyst powder in ethanol and applied on the carrier by use of a pipette. This sample pad of type S160 from Plano consists of a 10–15 nm thin carbon film, which is coated on a copper mesh 200. The samples were examined by use of a TEM Philips CM200 FEG and an acceleration voltage of 200 kV. The software Digital Micrograph was used to analyze the images. Scanning transmission electron microscopy (STEM) was performed on the Ir nanoparticles scratched from the MEA after NAP-XPS measurements. A LaB6-JEOL 2100 microscope operating at 200 kV and with a point to point resolution of 0.1 nm was used. The Fast Fourier Transformation (FTT) analysis was performed using the Digital Micrograph software.

### Electrochemical measurements and RDE preparation

All electrochemical measurements are performed in 0.5 M H_2_SO_4_ by a rotating disk electrode (PINE Research Instrumentation) method using a Metrohm Autolab PGSTAT12 in potentiodynamic mode. A platinum wire acts as the counter electrode and a reversible hydrogen electrode (HydroFlex by Gaskatel) as the reference electrode. The ohmic drop is determined by electrochemical impedance spectroscopy (EIS) making use of a second potentiostat (IM6 by Zahner-Elektrik).

To produce a catalyst ink 10 mg powder is added to 8.3 ml ultra-pure water and 40 μl Nafion resin being subsequently sonicated in an ice bath for 30 min. After brief electrode polishing with 0.05 μm aluminum suspension, 10 μl ink is drop-cast onto a glassy carbon disk (diameter of 5 mm) resulting in a loading of 6.1 × 10^–5^ g_Ir_ cm^–2^. Afterwards, the coating is dried under an inert gas atmosphere. Ir-black by Umicore is used as the reference catalyst.

Before starting the measurement the electrolyte (0.5 M H_2_SO_4_) is purged with N_2_ for at least 10 min. In the next step the coated electrode is mounted to the rotating unit and plunged into the sulfuric acid. All cyclic voltammetries (CV) are performed at a rotation speed of 2300 rpm and an electrolyte temperature of 25 °C. Moreover, the gas supply is not interrupted during measurements.

Table S1† shows the different protocols of CV applied consecutively to evaluate the OER activity (CV1), to clean the surface (CV2) and to determine the turnover frequency (TOF) from the Ir^III^/Ir^IV^ redox peak (CV3). The results are corrected by taking ohmic losses (IR drop) and capacitive current into account.

MEAs having Ir-black (Umicore) and Ir-nano 99.8 anodes were prepared, and tests in the PEM electrolyzer stack were performed in order to obtain additional information about the functionality and stability of the catalysts. The MEAs were elaborated using the wet spraying technique from suspensions with the catalysts having the same proportion of solvents and Nafion ionomer. The results are presented in Fig. S7.†

### *Operando* near ambient pressure X-ray photoelectron spectroscopy (NAP-XPS)

The XPS experiments were carried out at the NAP-XPS end station of the University Pierre et Marie Curie set on the TEMPO beamline^29^ of the SOLEIL synchrotron in France. The analysis chamber of the NAP-XPS is equipped with a SPECS Phoibos 150-NAP hemispherical electron analyzer including an electrostatic lens system with four differential pumping stages allowing a working pressure up to 20 mbar. A windowless beamline entrance with 3 differential pumping stages secures the pressure of the beamline below a limit of 5 × 10^–8^ mbar. The analyzer is equipped with a Delay Line detector from Surface Concept. The photon energy of the TEMPO beamline ranges from 40 to 1500 eV with a resolution better than 70 meV. The spectra presented here were collected with pass energy of 50 eV, giving an overall resolution better than 0.15 eV for all the photon energies used. The NAP-XPS chamber is also equipped with a quadrupole mass spectrometer in order to control the gas-phase composition of the chamber.

To study the electrocatalyst behavior under different polarization conditions the membrane electrode assemblies (MEAs) have been prepared by the catalyst-coated membrane method. The MEA preparation procedure was previously described.^30^ The studies of Ir-nano 99.8 electrodes were performed at 3 mbar oxygen-free water vapor pressure under a constant voltage applied between the working electrode (WE) and the counter electrode (CE), controlling the current values by means of chronoamperometry. The impedance spectroscopy measurements as well as other electrochemical techniques were performed using a μ-Autolab potentiostat from Metrohm. The example of the CV obtained in the NAP-XPS chamber is shown in Fig. 8a. The XP spectra of the corresponding elements were recorded using the kinetic energy of the emitted photoelectrons of *ca.* 530 eV. For the depth profiling analysis the Ir 4f XP spectra were collected using the following photon energies: 460 eV, 595 eV and 1080 eV. The corresponding inelastic mean free paths (IMFPs) of the emitted electrons were estimated using QUASES-IMFP-TPP2M software. The analysis depth was calculated as three times the IMFP and equaled 1.9, 2.3, and 3.6 nm, respectively. The binding energy (BE) values were calibrated with respect to the C 1s position (284.8 eV). The peak fitting of Ir 4f XP spectra was performed based on Pfeifer *et al.*^31^ The spin orbit splitting as well as the area ratio between the Ir 4f_7/2_ and Ir 4f_5/2_ components was constrained to 3 eV and 4 : 3, respectively. Shirley background was used for all the spectra. Curve fitting was performed based on a hybrid Doniach Sunjic/Gaussian–Lorentzian (sum) function; for the details the reader should referred to ref. 31. The assignment of Ir 4f components was done based on the literature data of the BE values summarized in Table S2 of the ESI.† The analysis of O 1s XP spectra cannot be carried out due to the presence of Si-based species, most probably SiO_*x*_ originating from the membrane.

### Quantitative modeling of XP spectra

To estimate the thickness of the formed Ir^IV^ oxide layer in the OER region, the Ir 4f XP spectra were simulated using the SESSA software (Version 2.0).^32^ The modeling was carried out with a “layered sphere” morphology (Fig. S5†), consisting of an outer Ir^IV^ oxide layer of various thicknesses (from 0 nm to 0.35 nm) and inner Ir metal. The particle diameter was maintained constant at a 2 nm value corresponding to a mean diameter of Ir nanoparticles. The photon energies were varied according to the experimental values (460 eV, 595 eV, 1080 eV) with the mean free paths calculated by the software. The results are shown in Fig. S6.†

## Discussion of results

### Morphology and nano-structure

**Fig. 2:**
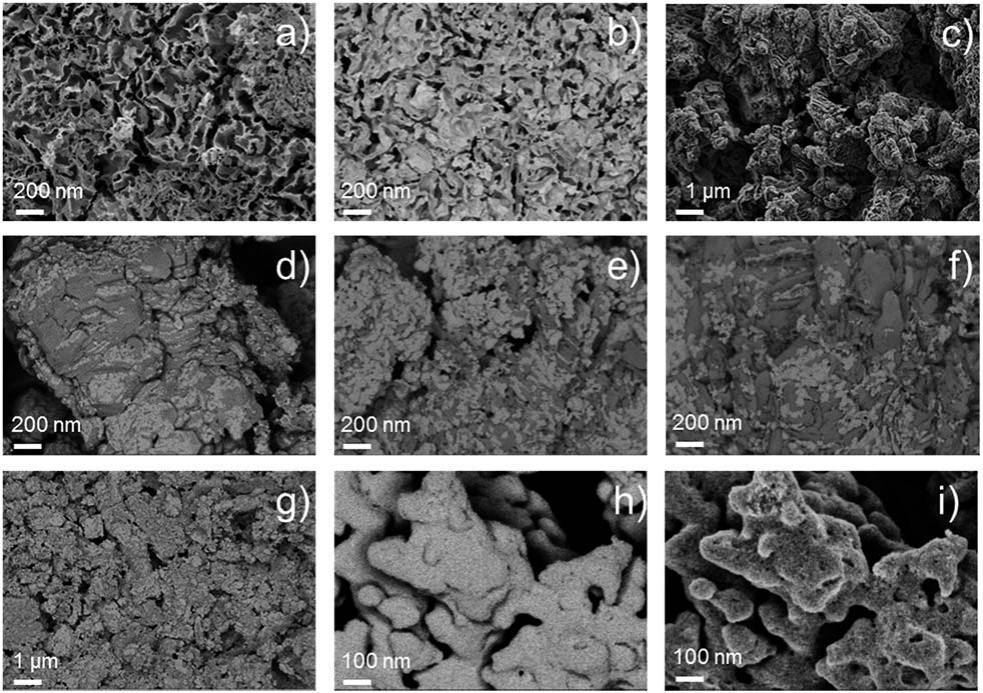
SEM images from the secondary and back-scattered electron signals (SE, BSE) of catalysts (a) SE Ir-black and (b) BSE Ir-black; (c) SE Ir-nano H_2_O; (d) BSE Ir-nano 91.5; (e) BSE Ir-nano 99.5\CTAB; (f) BSE Ir-nano 99.5; (g) BSE Ir-nano 99.8; and (h) BSE Ir-nano 99.8-P, (i) SE Ir-nano 99.8-P.

**Fig. 3:**
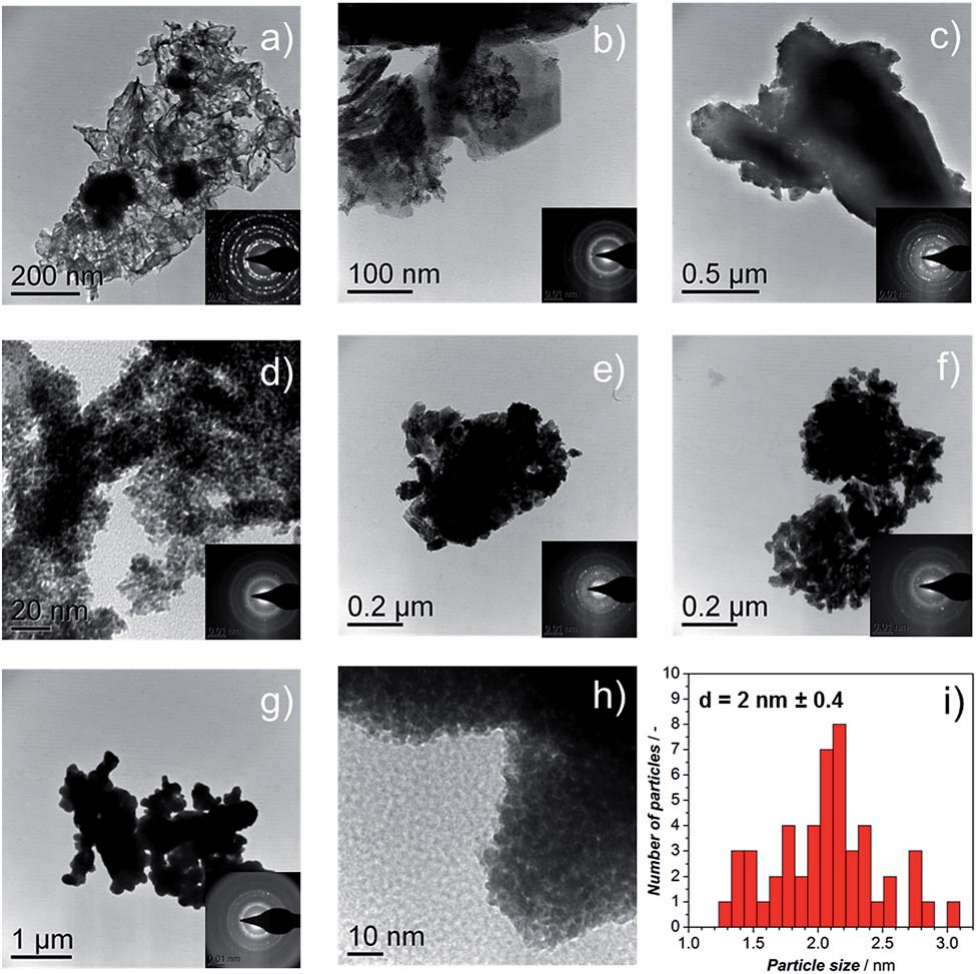
TEM images of catalysts (a) Ir-black; (b) Ir-nano H_2_O; (c) Ir-nano 91.5; (d) Ir-nano 99.5\CTAB; (e) Ir-nano 99.5; (f) Ir-nano 99.8; and (g), (h) Ir-nano 99.8-P; the insets show the electron diffraction pattern from the synthesized materials showing the (111), (002), (022), (311), and (222) reflections of metallic Ir. (i) Histogram of Ir-nano 99.8-P.

The morphology of the synthesized catalysts was studied by SEM. Micrographs recorded with the secondary electrons (SEs) revealed the topographic contrast of the different materials, while the back-scattered electrons (BSEs) allowed distinguishing the Ir nanoparticles from the traces of the IrCl_3_ precursor. The commercial Ir-black powder, Fig. 2a, showed a foam flake-shaped morphology with a large number of sheets, sharp corners and defects, which reflect the high surface area of the material. No impurities at the micro-scale could be observed from the BSE image of Fig. 2b. In contrast, the material synthesized in aqueous medium, Ir-nano H_2_O, shows particle agglomerates deposited on much larger crystals of another material (Fig. 2c), which could be clearly visualized by TEM and identified as unreduced IrCl_3_, *cf*. Fig. 3b and c.

XRD patterns on the synthesized materials clearly show that the large crystals shown in Fig. 3b–f correspond to IrCl_3_ (monoclinic, *C*2/*m*, 12). Furthermore, the intensity of the peak situated at 29.5° and 34.9°, which corresponds to the planes (130) and (131), respectively, is inversely proportional to the mass ratio Ir/Cl measured with EDX, which will be discussed later. These results have been included in the ESI (Fig. S0†).

The BSE images, Fig. 2d–g, of the catalysts synthesized with different EtOH purities clearly show the presence of IrCl_3_, which remained in the catalysts even after repetitive steps of cleaning with H_2_O. We assume that it is the high coverage of IrCl_3_ crystals with Ir nanoparticles, Fig. 2g, which hinders the dissolution of the salt. Adding NaBH_4_ in the synthesis pot in a 20-fold excess allows reducing the IrCl_3_ almost completely, Fig. 2h, producing a unique nano-porous catalyst with high rugosity, Fig. 2i.

TEM micrographs and electron diffraction (ED) patterns confirm the nano-structure of the synthesized materials. Fig. 3a–f show the TEM images of the Ir-black and Ir-nano materials, arranged in a similar order as in Fig. 2. The ED patterns are presented as an inset for each image. The first and the brightest ring of the ED corresponds to the (111) reflection, followed by (002), (022), (311), and (222) reflections of the face-centered-cubic (fcc) structure of metallic iridium.

As shown in Fig. 2d–g, the presence of large laminar IrCl_3_ crystals is visible for the material synthesized in H_2_O, Fig. 3b, their occurrence diminishing as either the purity of EtOH, Fig. 3c–f, or the amount of reducing agent, Fig. 3g and h, is increased. These results were confirmed by X-ray diffraction (XRD), Fig. S0.† Fig. 3i represents a typical Ir particle size distribution for the purest and cleanest sample, Ir-nano 99.8-P. Note that the average Ir particle size of 2 ± 0.4 nm showed little dependence on the sample purity.

**Fig. 4:**
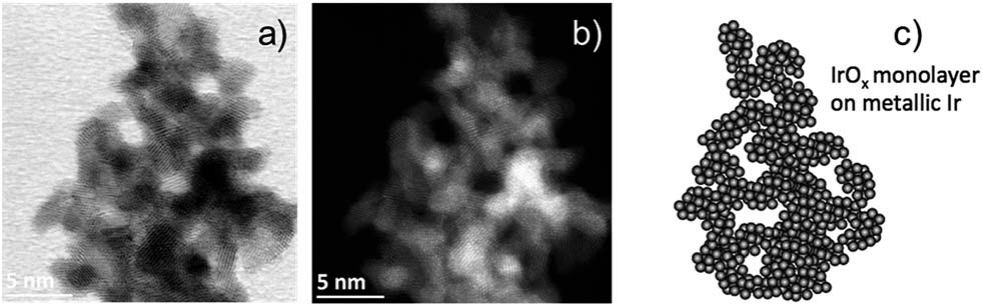
(a) and (b) STEM images of the Ir nanoparticles from the Ir-nano 99.8 electrode after the OER (performed in the chamber of the NAP-XP spectrometer). (c) Schematic illustration of the Ir-atoms forming nanoparticles interconnected based on the overall results from the physical characterization.

Post-mortem STEM analysis of the MEAs was carried out to determine the morphological changes in the Ir-nano catalysts after they were subjected to the OER. Fig. 4 shows a typical image acquired for the Ir-nano 99.8 electrode after operation. One may see that Ir nanoparticles are assembled in loose and highly porous agglomerates. This nanoporous morphology is preserved even after the electrochemical oxidation of the metallic core in the MEAs and is likely to be responsible for the high activity of the Ir nanocatalysts. The average diameter of the Ir nanoparticles remained approximately the same as that before electrochemical oxidation (Fig. 3). The core of the particles is metallic as confirmed by the Fast Fourier Transformation (FFT), which shows (111) and (200) reflections of fcc Ir. The reason for observing only these most intense reflections in the FFT is related to the small area of the analyzed spot (comprising 1–3 nanoparticles) in high resolution STEM, whereby the ED patterns were taken from lower resolution TEM images, and thus correspond to a much larger area.

### Oxygen evolution reaction (OER) activity

**Fig. 5:**
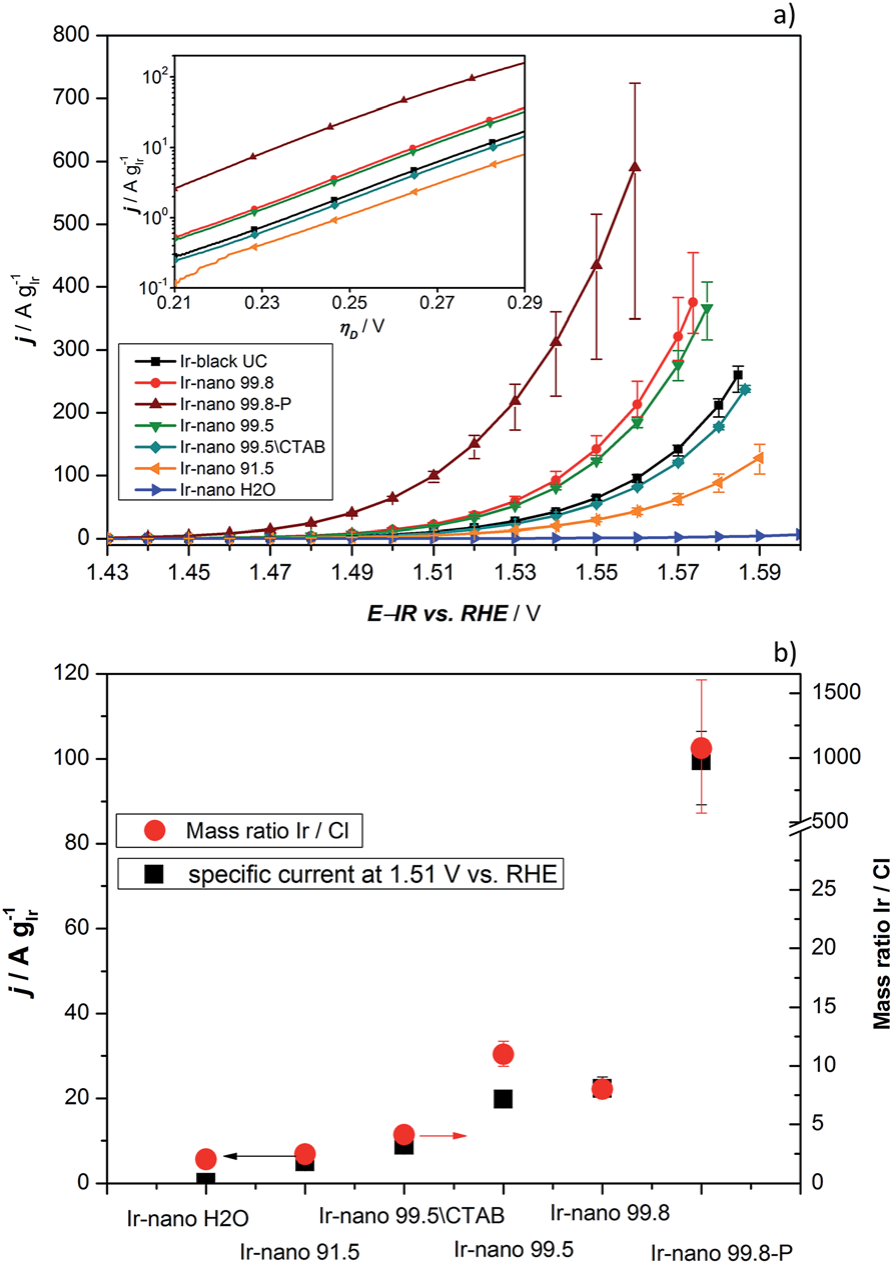
(a) OER activity of all compared catalysts. The scanning rate, temperature and rotation speed are 5 mV s^–1^, 25 °C, and 2300 rpm, respectively. The measurements were performed in 0.5 M H_2_SO_4_ and Ar-saturated solution; the inset shows the data as Tafel plots; (b) specific current density at 1.51 V *vs.* RHE measured under the same experimental conditions. The right axis indicates the Ir/Cl mass ratio recorded by EDX.

In order to investigate the influence of the different purities of ethanol to the point of replacement of the ethanol by water as well as the influence of the use of CTAB a thorough electrochemical characterization was performed. Fig. 5a presents the typical polarization curve for determining the activity of the different catalysts. As a commercial benchmark, Ir-black was used. Obviously, the use of ultra-pure water is not sufficient for the presented synthesis of OER catalysts and in what follows will not be considered due to its lack of performance. However, the presented figure shows the relationship of the purity of the ethanol and the activity for the OER. The synthesis with the cheapest ethanol and the purity of 91.5% also yields the catalyst with the lowest performance followed by the synthesis with EtOH 99.5 without CTAB which has apparently a positive impact on the catalyst performance. Certainly, the choice of the EtOH-quality achieves an optimum in the tradeoff between activity and costs. The activity of the Ir-nano catalysts synthesized with CTAB and EtOH 99.5, which is half as expensive as EtOH 99.8, the qualitatively best ethanol used, is within the error bar of Ir-nano 99.8 from our reference synthesis.

It is thus clear that the impurities in the solvent such as water react with the reducing agent, which justifies the need for adding it in a 4-fold excess during the synthesis. However, even this excess seems to be insufficient for a complete reduction of the precursor IrCl_3_ since Cl impurities may be detected even in the Ir-nano 99.8 sample. With decreasing the solvent purity, the proportion of the Cl increases (EDX analysis). Fig. 5b presents a relationship between the Ir/Cl mass ratio and the mass activity at 1.51 V *vs.* RHE. By increasing the amount of the reducing agent five-fold it was possible to increase the Ir/Cl mass ratio by three orders of magnitude, resulting in the mass activity of 100 A g_Ir_^–1^ at 1.51 V *vs.* RHE for Ir-nano 99.8-P, which is the highest OER activity so far reported for a pure Ir catalyst. This exceptional activity may be attributed to the small (*ca.* 2 nm) particle size of Ir nanoparticles, a unique nanoporous morphology, which is preserved even after the OER, as well as to the small thickness of the oxide layer on the surface of Ir nanoparticles (*vide infra*). The fact that lower purity Ir-nano samples show significantly lower activity may be attributed to the presence of unreacted IrCl_3_ crystals, which impede electron transfer from Ir nanoparticles during the OER.

Since we use variations of the same synthesis route we can directly compare electrochemical parameters for various samples. The overpotential *η*_A_ shown in Fig. 5a can be described as follows:^33^

**Fig. 6:**
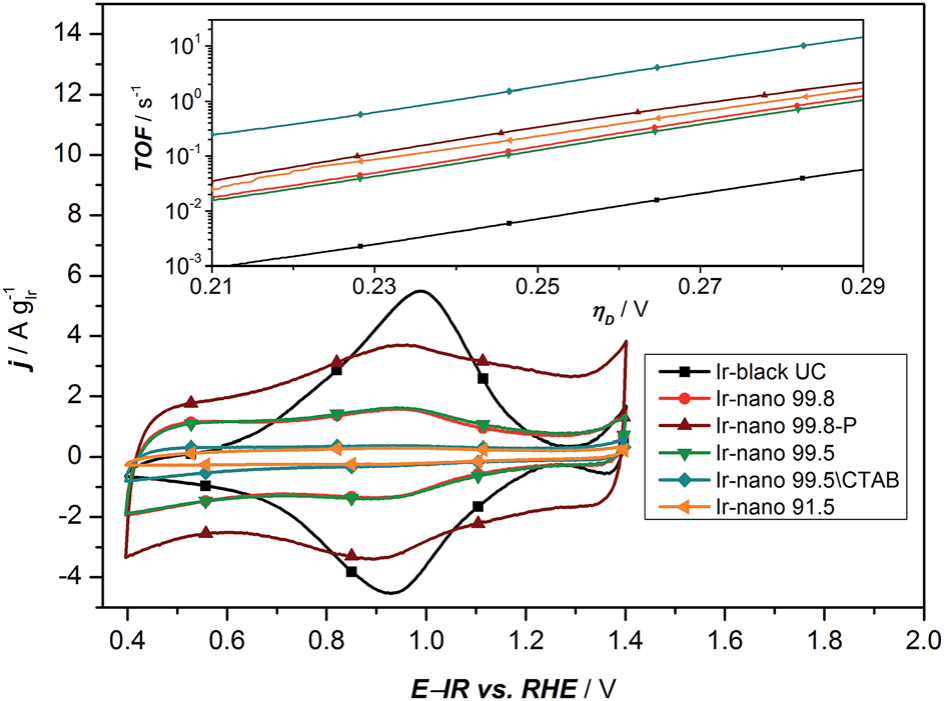
Cyclic voltammetry of all synthesized materials with different purities of EtOH. The inset presents the calculated TOF based on the analysis of the redox peaks of the CV.

1 *η*_A_ = (2.303*RT*/*αF*)log(*j*/*j*_0_)

where *T* is the temperature, *R* the ideal gas constant, *α* the apparent transfer coefficient, *F* is the Faraday constant (96 485 C mol^–1^), and *j* is the current density. The mass-specific exchange current density (*j*_0_, intercept at *η*_A_ = 0) and the Tafel slopes *b* can be determined from the linear parts of the curves represented in the inset of Fig. 5a (Tafel plots). Those electrochemical parameters, together with other important parameters which can be extracted from Fig. 5 and 6, are summarized in Table 2 and are worthy to be discussed. The Tafel slope was fitted for all catalysts with EtOH at overpotentials between 230 and 260 mV. The Tafel slopes and the corresponding apparent transfer coefficients *α* are close to the values of 40 mV dec^–1^ and 1.5, respectively. These correspond to the second electron transfer as the rate determining step of the OER mechanism for IrO_2_ according to the Krasil'shchikov path.^35^,^36^ The authors, however, pointed out that the graphical evaluation of the Tafel analysis can lead to misinterpretations of the specific exchange current density due to the nature of the graphical analysis and the extrapolation which are difficult to avoid. Therefore, due to the extrapolation to zero overpotential, small changes at the Tafel slope lead to unexpected behavior of the exchange current densities. The Tafel slope of the Ir-nano 99.8-P catalyst is in fact the closest to the theoretical value, but for the other catalysts, the impurities can affect the porosity of the electrode influencing the Tafel slope and *α*.^37^ Tafel slopes should be similar for all synthesis batches since the active component is assumed to be the same (*cf*. similar Ir particle size discussed above) and changes in the OER activity are related to the corresponding number of active sites. This supports the observation that Ir nanoparticles deposited on IrCl_3_ are not active due to poor electrical connection.

**Table 2:** Electrochemical parameters

Ink	*j*_1.48 V_ [A g_Ir_^–1^]	*j*_1.51 V_ [A g_Ir_^–1^]	*b* [mV dec^–1^]	*α* [—]	*j*_0_ × 10^–6^ [A g_Ir_^–1^]	*j*_cap_ × 10^–1^ [A g_Ir_^–1^]
Ir-black UC	2.2	10.4	42.9	1.38	3.16	3.55
Ir-nano 99.8	4.4	22.3	41.2	1.44	3.79	6.99
Ir-nano 99.8-P	24.2	99.6	39.7	1.49	13.50	26.74
Ir-nano 99.5	3.9	19.8	41.7	1.42	3.93	7.67
Ir-nano 99.5\CTAB	1.8	8.9	42.7	1.38	2.57	2.99
Ir-nano 91.5	1.1	5.1	46.4	1.28	4.48	2.12
Ir-nano H_2_O	0.1	0.3	63.8	0.93	9.92	—

Comparing the activities of the catalysts, presenting specific current densities at reasonable potentials such as 1.48 V, 1.51 V or 1.56 V *vs.* RHE is more meaningful as it is customary in the literature.^11^–^13^,^22^,^26^,^34^

As a characteristic footprint, CVs before the OER region and between 0.4 and 1.4 V *vs.* RHE demonstrate the first significant differences between the reference catalyst Ir-black and the synthesized Ir-nano catalysts. In Fig. 6, all CVs are presented. Even if all catalysts have similar physical properties and a metallic core surrounded by a thin oxide/hydroxide layer,^22^ as reported for the most recently published highly active Ir based catalysts,^11^,^18^,^22^,^38^ a distinctive redox peak of Ir^III^ to Ir^IV^ can mainly be observed for Ir-black. This redox peak can be used for calculating the turnover frequency (TOF) with respect to the overpotential,^11^,^22^,^39^ which is presented in the inset of Fig. 6 and calculated as follows.

2 TOF = *j*/(*zN*_s_*Q*_e^–^_)

Under the assumption that the OER occurs on the same sites, which are involved in the Ir^III^ to Ir^IV^ redox transition,^34^ the charge under the Ir^III^/Ir^IV^ redox peak can be considered as a measure of the number of active sites *N*_s_ multiplied by the electron charge *Q*_e^–^_. Due to the small redox peaks of the Ir-nano catalysts, the correct analysis of the TOF is challenging. These peaks are ill-defined probably due to a high surface heterogeneity and presence of slightly shifted peaks for various surface sites. The TOF for the synthesis without CTAB is unexpected but precisely the highest. This is most likely caused by an inaccurate analysis of an almost non-existing redox peak. The TOF of all synthesized materials is expected to be in the same range. Clearly divergent is the TOF of Ir-black due to the relatively low current density and the distinctive Ir^III^/Ir^IV^ redox peak, which indicates a large number of active sites. Obviously, the absence of the correlation with the charge of the redox peak and the activity suggests that this analysis method does not provide realistic information relative to the number of active sites. Therefore this method cannot be used for determining the active sites which are necessary for calculating the TOF.

**Fig. 7:**
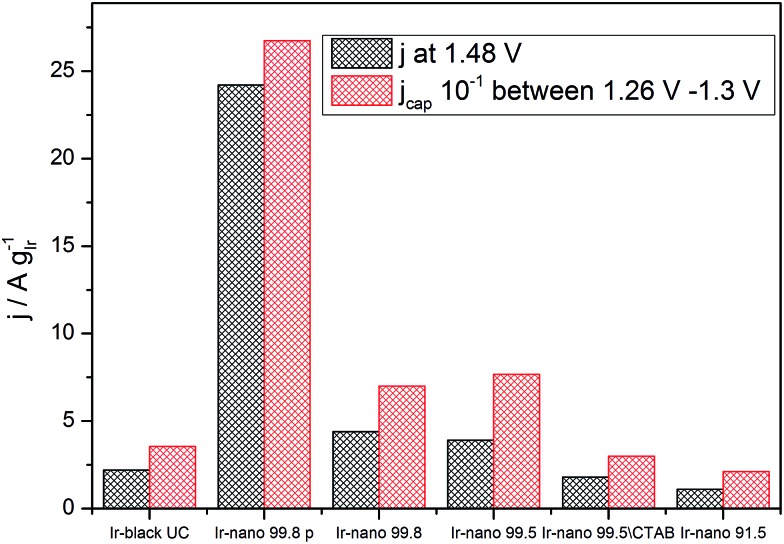
Correlation between specific average capacitive current and specific activation current density at 1.48 V (250 mV overpotential).

However, Fig. 7 presents the comparison between the OER activity and the capacitive current between 1.26 V and 1.3 V of Fig. 6, where no faradaic processes are expected.

The measured activity correlates well with the capacitive current (correlation coefficient: 0.994), suggesting that the latter provides a realistic access to the surface area, and that all samples have the same state of surface. Therefore the main reason for the high activity of the materials is the small particle size, yet the overall performance of the catalyst layer is greatly affected by the presence of IrCl_3_ impurities. Further information about the surface state of Ir nanoparticles during the OER will be provided by NAP-XPS measurements performed under *operando* conditions.

### *Operando* NAP-XPS analysis

**Fig. 8:**
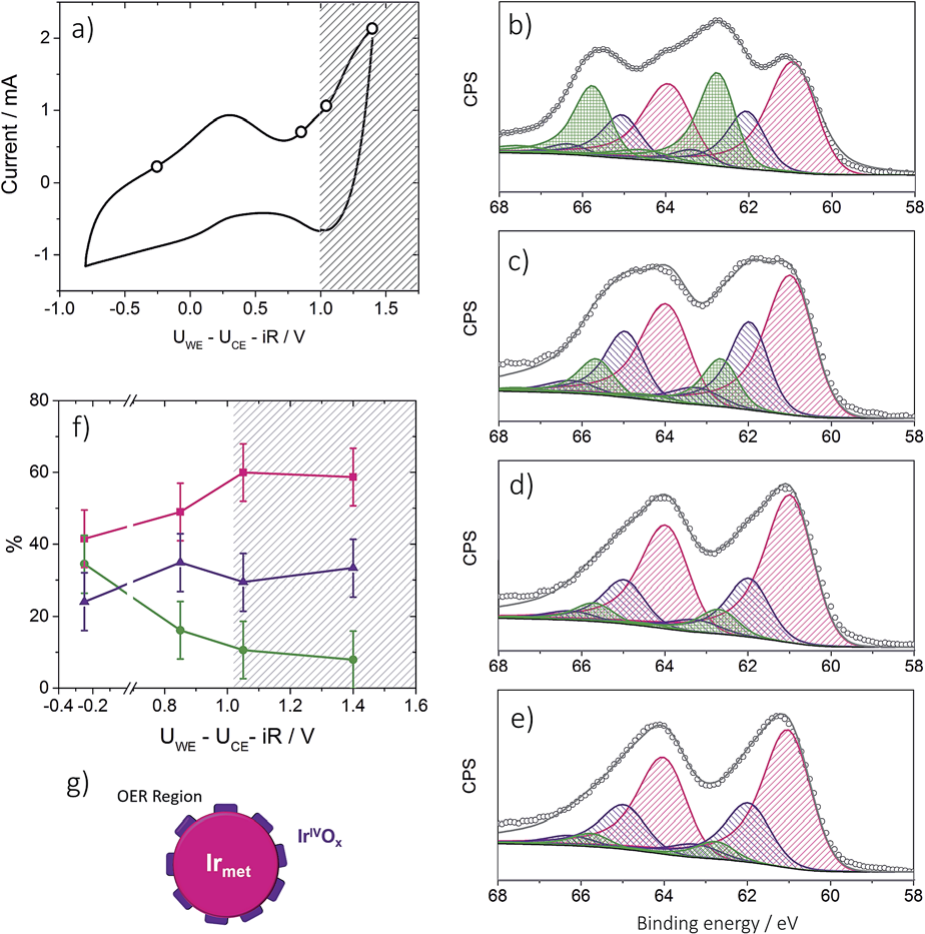
Panel (a): CV acquired in the analysis chamber of the NAP-XP spectrometer at 3 mbar water vapor pressure. Currents are plotted *vs.* ohmic drop corrected *U*_WE–CE_. (b)–(e): Ir 4f XP spectra of the Ir-nano 99.8 electrode obtained at 3 mbar water vapor pressure and under different polarization conditions (*U*_WE–CE_): –0.25 V (b); 0.85 V (c); 1.05 V (d); 1.4 V (e). Color codes: Ir_met_ (magenta); Ir^III^ (olive); Ir^IV^ (violet), fitted line (grey). The raw data are presented as open circles. Photon energy 595 eV; panel (f): potential dependence of Ir components: Ir_met_ (magenta); Ir^III^ (olive); Ir^IV^ (violet) for the Ir-nano 99.8 electrode. The hatched area corresponds to the OER region marked according to the MS data. Temperature 25 °C; panel (g): scheme illustrating a tentative structure of the IrO_*x*_ layer on the surface of Ir nanoparticles.

To provide insight into the exceptional OER activity of Ir-nanocatalysts compared to the state-of-the-art Ir-based materials we apply NAP-XPS and follow the surface composition of Ir-nano electrodes depending on the polarization. Considering similarities in the electrocatalyst synthesis and structure, only the Ir-nano 99.8 catalyst was investigated. The measurements were performed at ambient water vapor pressure in a two-electrode configuration with MEAs consisting of an Ir-nano anode (working electrode, WE) and a Pt/C cathode (counter electrode, CE; see Experimental). Before the NAP-XPS measurements, the surface of the Ir-nano 99.8 was stabilized by applying a few potential cycles in the interval of –0.25 V ≤ *U*_WE–CE_ ≤ 1.0 V, where *U*_WE–CE_ is the voltage applied between the WE and the CE. Fig. 8a shows a typical CV acquired in the measurement chamber of the XPS spectrometer. Compared to its counterpart acquired in a three-electrode liquid electrolyte cell, the CV is distorted, which may be attributed to a non-negligible overpotential at the CE. Nevertheless, it shows a characteristic anodic peak at *ca. U*_WE–CE_ = –0.25 V (commonly attributed to the Ir^III^/Ir^IV^ transition) and a current rise at high overpotentials, corresponding to the OER. Then, XP spectra were acquired under constant applied voltage (*U*_WE–CE_) of (i) –0.25; (ii) 0.85; (iii) 1.05 and (iv) 1.40 V. These values of the applied voltage correspond to (i) the potential interval below the anodic peak attributed to the Ir^III^/Ir^IV^ transition (*U*_WE–CE_ = –0.25 V); (ii) the potential region above the anodic peak (*U*_WE–CE_ = 0.85 V); (iii) the OER onset (*U*_WE–CE_ = 1.05 V) and (iv) the OER (*U*_WE–CE_ = 1.40 V). The OER was confirmed by the mass spectrometer integrated in the measurement chamber (the results are not shown) and the current transients shown in Fig. S1,† which demonstrate steady-state OER currents at *U*_WE–CE_ = 1.05 and 1.40 V.

Ir 4f XP spectra at different applied potentials are displayed in Fig. 8b–e. The fitting of the spectra was performed using three doublets with Ir 4f_7/2_ peak positions at 61.0, 62.1 ± 0.1, and 62.8 ± 0.1 eV, attributed to metallic Ir, Ir^IV^ and Ir^III^, respectively. In the case of Ir^III^ and Ir^IV^ satellite peaks at 64.6 ± 0.1 and 63.4 ± 0.1 eV, respectively, were used for fitting (see Table S2 for the BEs of reference compounds in the ESI†). The voltage dependence of the contributions of various surface species is shown in panel (f) of Fig. 8. One may see that the Ir-nano electrode subjected to CV cycles shows both oxide (Ir^III^ and Ir^IV^) and metallic species. Furthermore, by applying *operando* XPS, under the potential control and during the OER, we were able to observe the changes in the oxidation state of Ir with the applied potential. Indeed, one may clearly see that at potentials below the anodic peak in the CV of Fig. 8a, all three components, namely Ir_met_, Ir^III^ and Ir^IV^, are present in the XP spectrum, whereas increasing the cell voltage above 0.85 V leads to the disappearance of Ir^III^ sites such that in the OER potential interval the surface and the near-surface region of the electrode only contains metallic Ir and Ir^IV^ oxide (the contribution of the Ir^III^ species above 1.0 V falling below the uncertainty level of XPS as one can see from Fig. 8f). Two different Ir-nano-based MEAs were studied and showed similar behavior (see Fig. S3, ESI†), confirming the reproducibility of the measurements and the data analysis.

Tuning the photon energy (see Fig. S3†) resulted in small changes in the contributions of metallic Ir, Ir^IV^ and Ir^III^, thus not supporting a core–shell morphology and rather suggesting that oxidation of Ir nanoparticles results in the formation of a thin porous Ir^III^/Ir^IV^ layer on the surface. Once Ir^III^ is oxidized, the surface of Ir nanoparticles is dominated by Ir^IV^. The estimation of the Ir oxide thickness in the OER region (*U*_WE–CE_ = 1.40 V) was done using the SESSA software, simulating the XP spectra for the layered sphere morphology (see the ESI†). Note that we only considered one type of Ir oxide, since as shown above, Ir^III^ was fully converted into the Ir^IV^ oxide during the OER (*vide supra*). Comparing the experimental and theoretical results, the apparent oxide thickness was estimated as *ca.* 0.2 nm, which is comparable to a monolayer thickness. Considering all of the above we conclude that the Ir-nano catalyst, which is stabilized by potential cycling, consists of a metal core covered by a thin porous Ir oxide/hydroxide layer. The composition of this oxide layer strongly depends on the applied potential and changes from a mixed Ir^III^/Ir^IV^ oxide/hydroxide below the OER onset to an Ir^IV^ oxide monolayer above the OER onset, which further persists in the OER potential interval. This conclusion is in disagreement with the recent work of Minguzzi *et al.*,^34^ who applied *in situ* X-ray absorption spectroscopy (XAS) at the Ir–L_III_ edge to investigate changes in the redox state of an IrO_*x*_ film prepared by chemical oxidation of IrCl_3_ by H_2_O_2_ and concluded on the co-existence of Ir^III^ and Ir^V^ during the OER. Note however that XPS is more sensitive to the changes in the oxidation state, and is also highly surface sensitive. The high surface sensitivity is confirmed by the data shown in Fig. 8 (panels b–e) whereby even a submonolayer coverage of Ir^III^ oxide/hydroxide can be clearly observed and followed as a function of the applied voltage. Thus, our data suggest that Ir^III^ is absent from the particle surfaces under the OER conditions, and Ir^III^ is oxidized to Ir^IV^ below the OER onset. The Ir^III^/Ir^IV^ redox transition can be associated with the anodic peak observed at *U*_WE–CE_ = 0.25 V in the CV of Fig. 8a. This observation supports previous electrochemical studies, whereby Ir^III^/Ir^IV^ transition was postulated but not spectroscopically confirmed *in situ*.^24^,^25^,^40^–^42^

An intriguing and highly debated question refers to the formation of Ir in oxidation state(s) above IV. Formation of Ir^V^ under the OER conditions was postulated in early publications of Koetz^43^,^44^ *et al.* and Pickup and Birss.^41^ Recently Casalongue *et al.*^38^ based on their NAP-XPS study suggested formation of Ir^V^ during the OER on IrO_2_ oxide. This conclusion has been challenged by Pfeifer *et al.*^31^,^45^ who combined XPS measurements with DFT calculations to show that, due to the final state effects, the high BE component rather corresponds to Ir^III^ species, and attributed the peaks observed in cyclic voltammetry at the OER onset to an anion O^II–^/O^I–^ rather than cation redox transition. Note also that the lack of either reliable Ir^V^ reference data or theoretical calculations of the BE shifts for Ir^V^ compounds complicates the debate around the formation of Ir OER intermediates in high oxidation state(s) (V or higher). While our recent work on mixed IrRu performed using *operando* NAP-XPS^30^ did not confirm formation of any higher (than IV) oxidation states of Ir in the OER interval, one could argue that it was due to a relatively high (530 eV) kinetic energy of photoelectrons. In this work we decreased the incident photon energy down to 460 eV close to that used in ref. 38 (corresponding to Ir 4f photoelectrons with kinetic energy of *ca.* 395 eV) but did not observe appearance of any additional peaks in the Ir 4f BE interval during the OER. Thus, this work does not support formation of Ir species in oxidation states different from IV under the OER conditions.

Another question concerns the nature of the Ir^IV^ species formed on the surface of Ir nanoparticles during the OER. Even if the XPS is sensitive to the composition rather than the structure, analysis of the peak width of the Ir 4f doublet corresponding to Ir^IV^ for the reference IrO_2_ samples with a rutile structure (see spectra in Fig. S2†) suggests that the Ir^IV^ oxide film formed on Ir nanoparticles in this work is most likely amorphous rather than crystalline. Indeed, Pfeifer *et al.*^31^ demonstrated that crystalline and amorphous Ir^IV^ oxides are characterized by different XP peak widths (FWHM). Formation of amorphous Ir^IV^ oxide is in agreement with the monolayer thickness of the oxide film on metal Ir particles in this work. Thus, we conclude that the surface and subsurface composition of the Ir-nano electrode differs significantly from that observed for the rutile-type IrO_2_.^30^ Indeed, Ir-nano consists of metallic Ir covered by a thin layer of Ir^III/IV^ (hydro)oxide below the OER onset, which is transformed into a monolayer of (amorphous) Ir^IV^ oxide in the OER interval, while XP spectra of thermally obtained IrO_2_ oxide nanoparticles conform with crystalline rutile-type IrO_2_. It is interesting to note that the observed decrease of the contribution of the Ir^III^ component above *U*_WE–CE_ = 1.05 V is not accompanied by an increase in the contribution of the Ir^IV^ component expected for an Ir^III/IV^ redox transition. Instead, the Ir^IV^ component rests fairly independent of the applied voltage, while the Ir metal component increases slightly with the applied potential, which is counterintuitive (Fig. 8f). This experimental observation could be either attributed to the dissolution of Ir surface oxides, as previously reported by Cherevko *et al.*,^46^ or to their transformation involving either changes of the oxide density or morphology. The former explanation seems unlikely on the time scale of our measurements considering the stable PEM electrolyzer operation of the prepared anodes for at least 100 h (Fig. S7†). Moreover, we have previously demonstrated in a commercial PEM electrolyzer that the degradation rates of MEAs with non-thermally oxidized Ir are sufficiently low for allowing long-term operation.^47^,^48^ Further stability tests with the developed catalysts are part of our ongoing work.

The XPS data do not allow distinguishing between the oxide thinning due to the transformation of hydrous into an anhydrous oxide or formation of a porous Ir^IV^ oxide layer at high anodic potentials from a more compact Ir^III/IV^ film at lower potentials. The latter seems however more likely and is in good agreement with reports on Ir-black and Ir polished disc electrodes, which become porous under redox cycling, providing access to the metal underneath the surface oxide.^23^,^40^ Fig. 8g provides an illustration of the tentative morphology of Ir/IrO_*x*_ nanoparticles. The different surface composition/structure of Ir-nano compared to stoichiometric rutile-type IrO_2_ accounts for the significantly higher activity of the former.

Finally, the exceptionally high activity of Ir-nano 99.8-P compared to commercial Ir-black or other commercial Ir nanoparticles^49^ can be attributed to their nano-porous structure. Indeed, it has been reported that nano-porous dendritic structures of Ir supported on Vulcan XC-72 show 3-fold higher OER activity^13^ than Ir nanoparticles with similar particle size, *ca.* 2 nm,^23^ uniformly dispersed on the same support. Similarly, in PEM fuel cells it has been found that, after Ni leaching, the Pt-rich Pt_3_Ni polyhedra (nano-frames) supported on carbon are much more active for the oxygen reduction reaction (ORR) than Pt_3_Ni/C.^50^,^51^ The leaching of an alloying element in the IrO_x_ structure also leads to a substantial enhancement in catalytic activity.^18^,^52^ The STEM images of Fig. 4a and b show clearly a similar nano-porous morphology of unsupported Ir nanoparticles. Consequently, the fact that the Ir-nano material displays a 10-fold higher OER activity compared to that of the flake-like structured Ir-black cannot be attributed only to an increase in the surface area but is likely related to the nano-porous (nano-frame) structure of the Ir-nano catalyst. Similar to the Pt_3_Ni nano-frames, it is likely to be the large number of low-coordinated catalytic sites, corners, edges and defects in the Ir structure of Fig. 4 that lead to an unprecedented high atomic utilization. In this regard, this work fosters the scientific community to further investigate complex Ir-nanostructures with atomic level and *in situ* advanced spectroscopic techniques along with theoretical simulations.

## Conclusions

In this study, the influence of varying the chemical purity and the concentration of the reducing agent on the electrocatalytic activity of synthesized Ir-nano particles is thoroughly explored.

The use of a tensioactive agent (in this case CTAB) is as important as the use of high purity solvents and the amount of reduction agent in excess. However, there is scope for cost reduction in the synthesis procedure with ethanol (99.5%), which is half as expensive as 99.8% pure ethanol, resulting in similar electrocatalytic activity. Adding the reducing agent in excess enables almost complete elimination of Cl impurities in the final catalyst product and achieving an OER activity of 100 A g_Ir_^–1^, measured for the first time at 1.51 V *vs.* RHE.

The surface characterization of the Ir-nano electrode in the OER region performed by *operando* NAP-XPS revealed that electrochemical oxidation of Ir nanoparticles results in the formation of a monolayer-thick Ir oxide layer on the surface of metal particles. We also conclude that the Ir^III^/Ir^IV^ transition occurs below the OER onset, and under the OER conditions the surface is dominated by a thin amorphous Ir^IV^ oxide layer. Thus, the superior activity of Ir-nano compared to thermally oxidized IrO_2_ catalysts may be attributed to the small (*ca.* monolayer thick) thickness of the oxide film and its interaction with the underlying metal core. The fact that we do not observe Ir^V^ species during the OER suggests that either they are not formed, or their coverage under steady-state measurement conditions is below the detection limit. In the future it might be interesting to perform time-resolved measurements in order to clarify whether Ir^V^ or other Ir species with higher oxidation states are formed during the OER as short-lived intermediates.

## Conflicts of interest

There are no conflicts of interest to declare.

## Supplementary Material

Supplementary information
