# Supplementary material for: Highly active nano-sized iridium catalysts: synthesis and operando spectroscopy in a proton exchange membrane electrolyzer
Source: Chem Sci. 2018 Feb 20;9(14):3570–9. doi: 10.1039/c8sc00555a (PMC5934821; doi:10.1039/c8sc00555a)
Supplement: Supplementary file 1 [file SC-009-C8SC00555A-s001.pdf]

Electronic Supplementary Material (ESI) for Chemical Science.
This journal is © The Royal Society of Chemistry 2018

Supporting Information

Highly Active Nano-Sized Iridium Catalysts: Synthesis and Operando Spectroscopy in Proton Exchange Membrane Electrolyzer

P. Lettenmeier[a], J. Majchel[a], L. Wang[a], V. A. Saveleva[b], S. Zafeiratos[b], E. R. Savinova[b], J.-J. Gallet[c,d], F. Bournel[c,d], A. S. Gago[a,*] , K. A. Friedrich[a,e]

[a]Institute of Engineering Thermodynamics, German Aerospace Center (DLR), Pfaffenwaldring 38-40, Stuttgart, 70569, Germany

[b]Institut de Chimie et Procédés pour l'Energie, l'Environnement et la Santé, UMR 7515 du CNRS-Université de Strasbourg, 25 Rue Becquerel, 67087 Strasbourg, France

[c]Laboratoire de Chimie Physique-Matière et Rayonnement, Sorbonne Universités – UPMC Univ Paris 06 – CNRS, 4 place Jussieu, 75005 Paris, France

[d]Synchrotron-Soleil, L'orme des Merisiers, Saint Aubin – BP48 91192 Gif-sur-Yvette Cedex , France

[e]Institute of Energy Storage, University of Stuttgart, Stuttgart, Pfaffenwaldring 31, 70569, Germany

*E-mail: aldo.gago@dlr.de (A. S. Gago)

Physical characterization

X-ray diffraction (XRD) on Ir-nano 99.8, Ir-nano 99.5, Ir-nano 99.5\CTAB, Ir-nano H2O catalysts. The results are presented below. It is now clear that the large crystals shown in Figure 3b – 3f corresponds correspond to $IrCl_3$ (monoclinic, C2/m, 12). Furthermore, the intensity of the peak situated at 29.5° and 34.9°, which corresponds to the planes (130) and (131), respectively, is inversely proportional to the mass ratio Ir/Cl measured with EDX (Figure 5b).

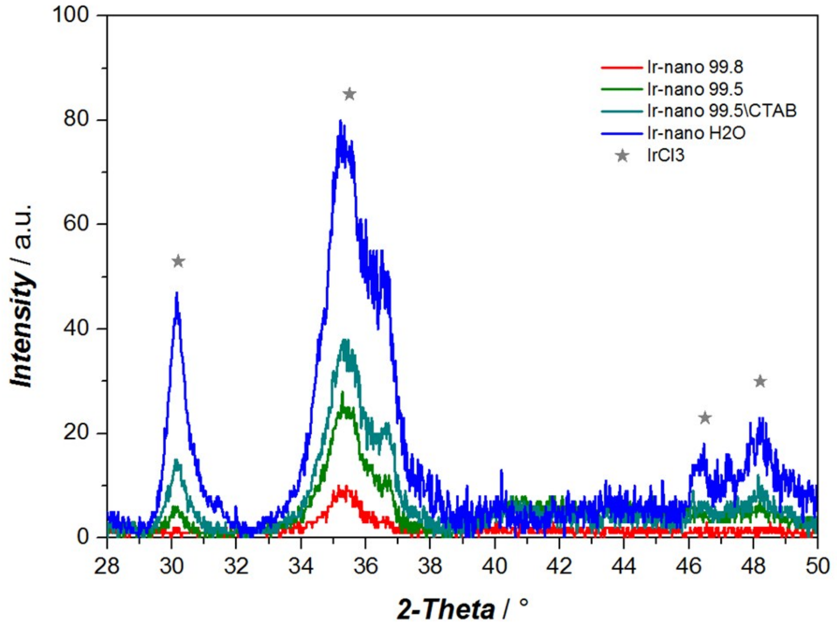

Figure S0: XRD patterns of Ir-nano 99.8, Ir-nano 99.5, Ir-nano 99.5\CTAB, Ir-nano H_2O catalysts. X-ray diffraction data were collected by using a D8 Discover GADDS diffractometer with VÅNTEC-2000 areal detector. The X-ray source (Cu-Kα) consisted of a tuned monochromatic and parallel X-ray beam (accelerating voltage: 45 kV, tube current: 0.650 mA). The samples were measured on reflection mode in four frames with θ1= θ2 (180 s per frame) and a step size of 2θ = 23° (first frame θ = 12°)."

Electrochemical characterization in 0.5 M H_2SO_4

The characterization protocol for the RDE measurements of all catalysts is summarized in Table S1.

Table S1. Protocol of cyclic voltammetric (CV) curves for the electrochemical characterization

CV	Potential, V vs. RHE	Scanning rate, mV s^{-1}	Number of cycles
1	1.00 – 1.60	5	3
2	0.05 –1.50	500	50
3	0.40 – 1.40	20	3

Electrochemical characterization of the MEA in the NAP-XPS chamber

The MEAs were prepared by the catalyst-coated membrane method. The catalyst inks containing Ir-nano 99.8 catalyst for WE side and HiSpec4000 Pt/C (Johnson Matthey) for the CE mixed with 20

wt.% of the ionomer was sprayed directly to the Aquivion (Solvay™). For more details the reader is referred to reference.[1] The measurements were performed in the chamber of NAP-XP spectrometer at 25°C under 3 mbar oxygen-free water vapor ambient. The MEA resistance determined by high frequency impedance spectroscopy before and after the NAP-XPS measurements was equal to 30 Ohm. This value was used to perform the Ohmic drop (iR) correction. NAP-XPS measurements were performed under constant voltage applied between the WE and the CE. The spectroscopic measurements were performed after the stabilization of the current values (ca. 2-3 min after the potential application) Current transients at selected voltage values are represented in Figure S1.

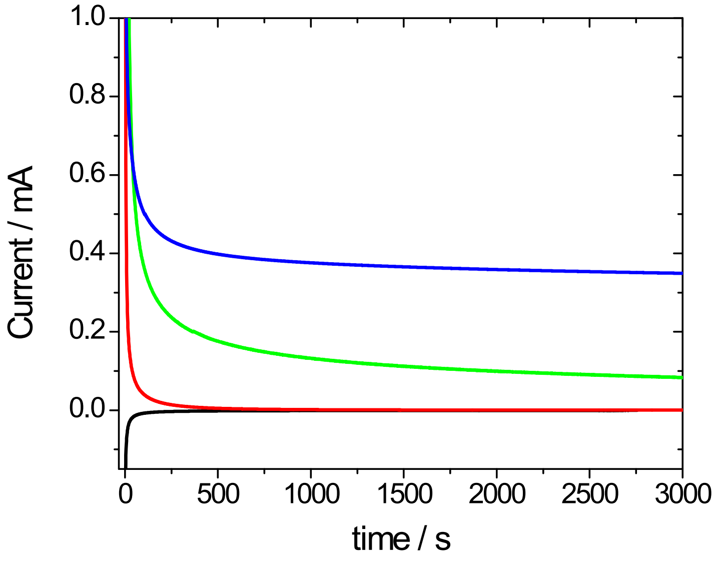

Figure S1. Current transients for MEAs with a Ir-nano 99.8 anode under 3 mbar water vapor and different polarization conditions (U-iR): -0.25 V (black); 0.85 V (red); 1.05 V (green); 1.40 V (blue).

Reference binding energy (BE) values for Ir species

Literature BE values for various Ir species are shown in Table S1. It should be noted that due to final state effects Ir(III) is characterized by higher BE values BE values compared to Ir(IV) species (see Table S1 and references therein). Rutile-type IrO_2 oxide powder was prepared by thermal oxidation of Ir nanoparticles at 490°C under the air and used as a reference (see Figure S2). The fitting procedure for IrO_2-rutile was based on the method reported elsewhere.[2] The analysis of the XP spectra revealed some differences between the rutile-type IrO_2 and electrochemical oxide formed on the surface of Ir nano catalyst in terms of the BE (61.8 eV vs. 62.3 eV) and the full width at half maximum (1.0 eV vs. 1.3 eV) suggesting that the oxide formed electrochemically on Ir nano particles most likely has an amorphous structure. Note however that XPS is more sensitive to the composition rather than the structure.

Table S2. Literature BE values for Ir species

Sample	Binding energy Ir $4f_{7/2}$ / eV			Reference
	Ir^0	Ir^{III}	Ir^{IV}	
Ir electrode<br>Molecular orbital calculations		62.1	61.6	[3]
Ir metal<br>$IrCl_3$	60.9	 62.6		[4]
$IrCl_3$<br>IrO_2 (thermally oxidized)		62.1	 61.5	[5]
IrO_2 (rutile)<br>IrO_x (amorphous)		 62.4	61.8<br>61.8	[2]
IrO_2 (bulk oxide)	60.9		62.1	[6]
IrO_2 NPs (thermal oxide)	60.8		61.7	[7]
Ir foil<br>Ir electrode (electrochemically oxidized)<br>IrO_2 (thermally oxidized)	61.1		 62.9<br>62.5	[8]
**Ir-nano 99.8**	**61.0**	**62.8**	**62.1**	**This work**
**IrO_2 rutile (thermal oxide)**			**61.8**	**This work**

Table S3. Binding energy positions of $Ir4f_{7/2}$ component of the doublets used in the fitting of Ir4f XP spectra.

**Component**	**Binding energy position of $Ir4f_{7/2}$ / eV**			
	**$U_{WE} - U_{CE}$ - iR = -0.25 V**	**$U_{WE} - U_{CE}$ - iR = +0.85 V**	**$U_{WE} - U_{CE}$ - iR = +1.05 V**	**$U_{WE} - U_{CE}$ - iR = +1.4 V**
Ir metallic	61.0	61.0	61.0	61.0
Ir (III)	62.8	62.7	62.7	62.8
Ir (III) satellite	64.6	64.5	64.5	64.6

Ir (IV)	62.1	62.0	62.0	62.0
Ir (IV) satellite	63.4	63.3	63.3	63.3

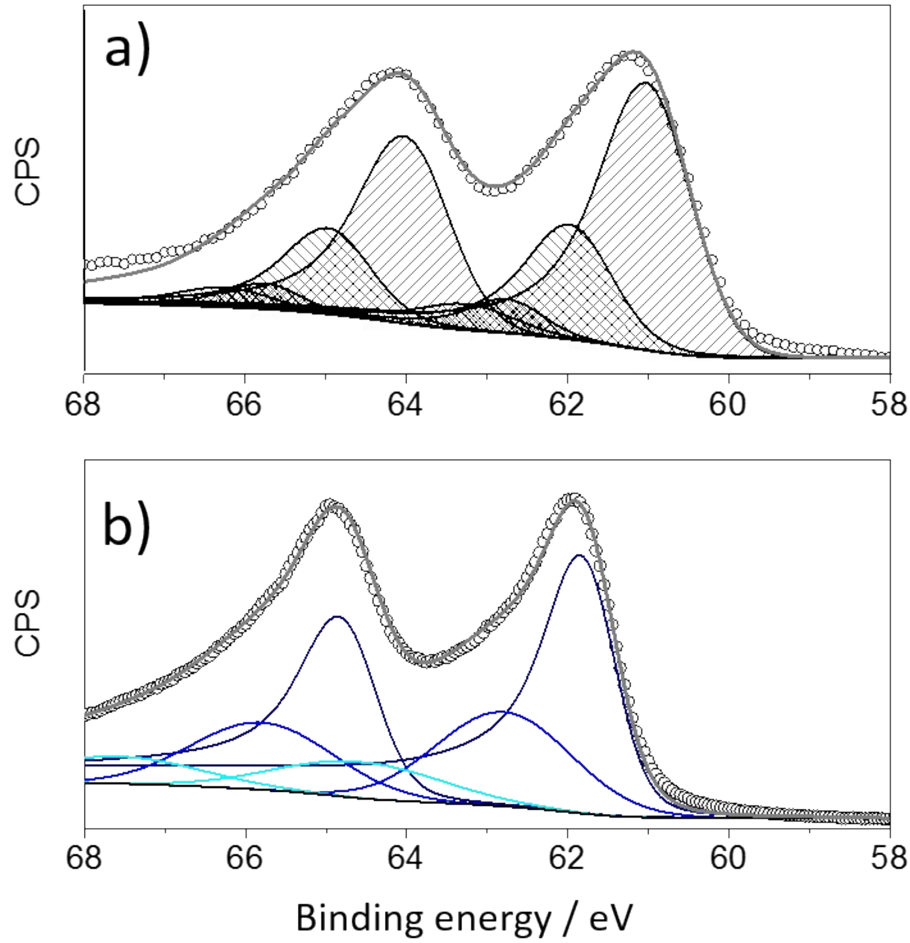

Figure S2. Ir4f XP spectra of (a) Ir-nano 99.8 electrode obtained under 3 mbar water vapor and U_{WE-CE} = 1.4 V and (b) rutile-type IrO_2 powder measured at the same station under UHV conditions. Color codes for panel (a) : Ir met (coarse); Ir III (cross); Ir IV (reverse coarse); for panel (b): Ir IV (dark blue); Ir IV sat 1 (blue); Ir IV sat 2 (cyan). The raw data are presented as open circles and the fitted data as a grey line. Incident photon energy 595 eV.

Reproducibility of NAP-XPS measurements

In order to confirm the reproducibility of the obtained results, the NAP-XPS measurement protocol was applied to a second MEA containing a similar Ir-nano 99.8 electrocatalyst at the anode. The presence of metallic Ir, Ir^{III} and Ir^{IV} components was observed and their potential dependence is shown in Figure S3, reflecting the same trends as those discussed in the main text of the manuscript.

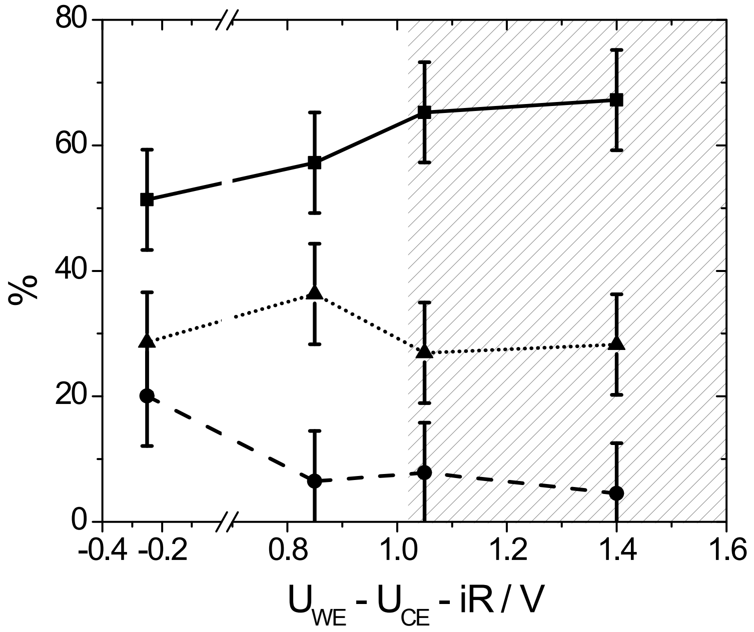

Figure S3. Potential dependence of Ir components for the 2nd Ir-nano 99.8 electrode: metallic Ir (solid); Ir^{III} (dashed); Ir^{IV} (dotted line). The hatched interval corresponds to the OER region defined using the MS data. Incident photon energy 595 eV.

NAP-XPS measurements. Depth profiling

One of the advantages of the synchrotron radiation is the ability to tune the photon energy allowing one to vary the depth of the analyzed sub-surface region. The incident photon energies used in this work are: 460, 595 and 1080 eV, which correspond to 1.9, 2.3 and 3.4 nm depth (estimated as three times the inelastic mean free path), respectively. The contributions of metallic Ir, Ir^{III} and Ir^{IV} determined with various photon energies are shown in Figures S4A-C as a function of the applied voltage. One may see that the contributions of the three components show little dependence of the photon energy. Such a behavior does not support a core-shell morphology but rather suggests that oxidation of Ir nanoparticles results in an inhomogeneous porous (hydr)oxide layer.

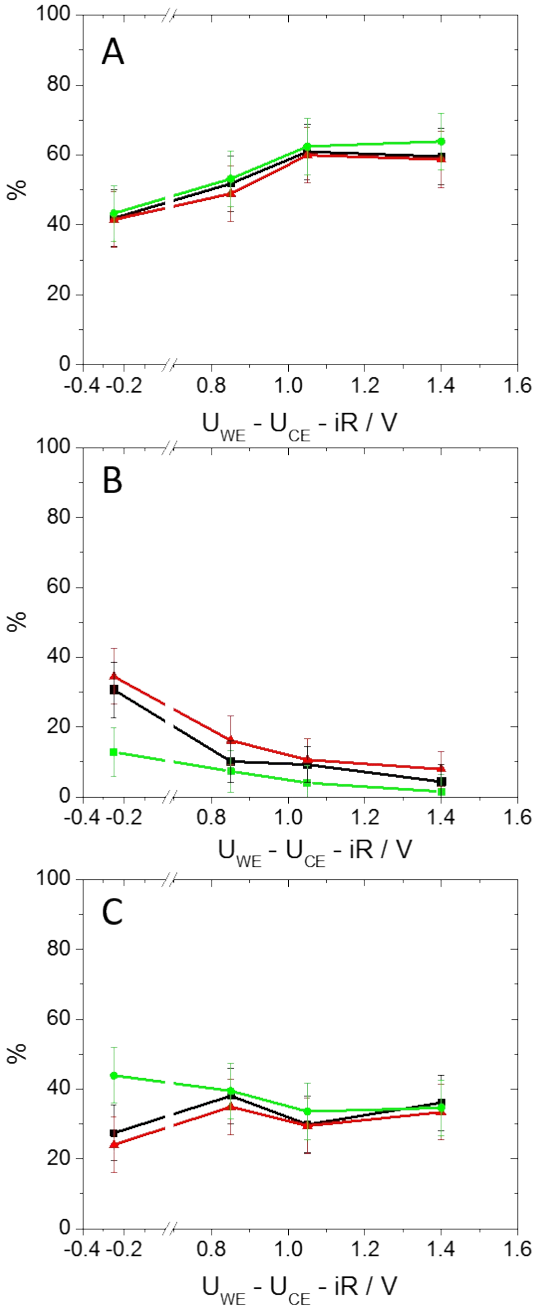

Figure S4. Fraction of Ir components: metallic Ir (A); Ir^{III} (B); Ir^{IV} (C) versus applied voltage at different incident photon energies: 460 eV (black); 595 eV (red); 1080 eV (green).

Simulation of Ir4f XP spectra

To estimate the thickness of the oxide shell on the surface of electrochemically oxidized Ir nanoparticles the SESSA software was utilized. Figure S5 shows the morphology used for the simulations.

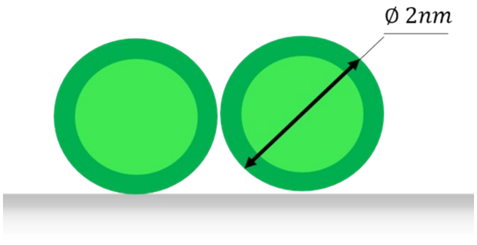

Figure S5. Sketch of the layered sphere model used for Ir4f XP spectra simulation with the SESSA software.

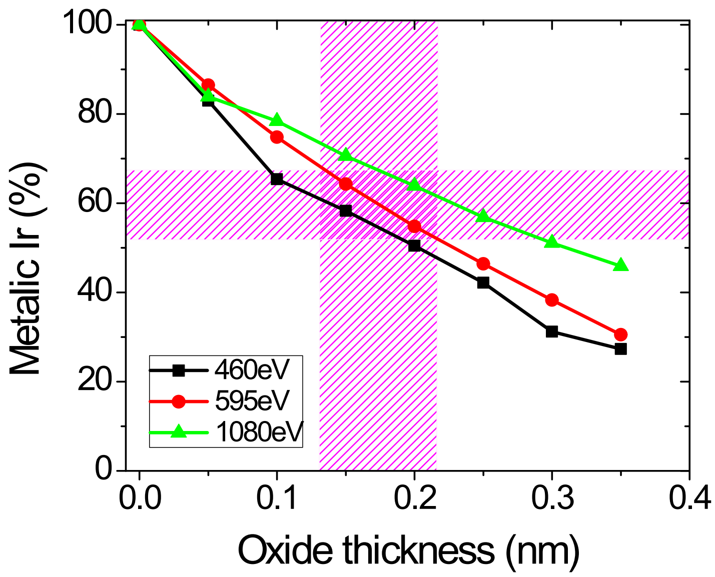

Figure S6. Calculated dependence of the metallic Ir contribution versus the oxide thickness for three different incident photon energies: 460 eV (black); 595 eV (red); 1080 eV (green). The hatched regions correspond to the experimental data for the photon energy of 595 eV and respective thickness values.

MEA tests in PEM electrolyzer

PEM electrolyzer constant operation tests at 1 and 2 A cm^{-2}, with MEAs having Ir black (Umicore) and Ir-nano 99.8 anodes were performed. The evolution of cell voltage with respect to time is presented in Figure S7. One can observe that the cell potential of Ir-nano 99.8 is slightly lower than the one with Ir-black, which can be attributed to the improved OER activity of the synthesized catalyst. However, the enhancement in activity cannot be one-to-one correlated with the RDE results presented in Figure 5a of the main text. The MEA manufacture greatly depends on the coating technique, ionomer content, hot-pressing pressure time/temperature, and numerous other engineering parameters. Therefore, it cannot be expected that these parameters should be the same for manufacturing MEAs with catalysts having large differences in OER activity between them. Yet, the PEM electrolyzer operation in both cases is constant during the measured time scale, even after spontaneous PEM electrolyzer system shutdowns. These initial durability tests demonstrate that the MEA with synthesized Ir-nano 99.8 at least is not less stable than the benchmark MEA commercial Ir-black catalyst.[9] Longer duration tests with high performance MEAs are imperatively necessary for commercial applications of the catalyst developed in this work.

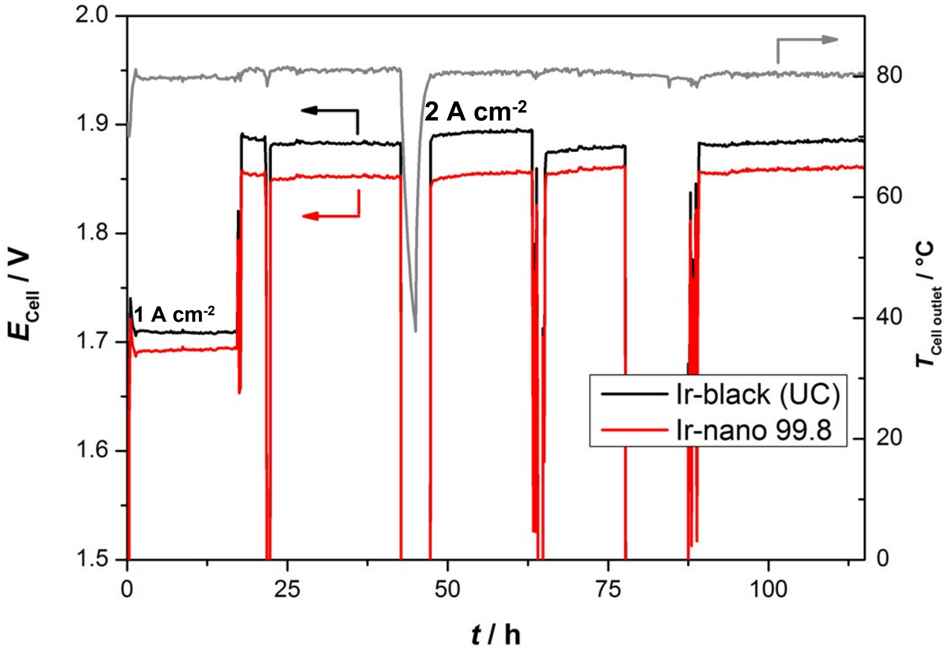

Figure S7. Two-cell stack with Ir-black (1 mg_{Ir} cm^{-2}) and Ir-nano 99.8 (1 mg_{Ir} cm^{-2}) with 25 cm^2 active cell area operating at 1 A cm^{-2} and 2 A cm^{-2}, 80 °C, 1 bar for 100 h. Nafion 212 was used as PEM.

References

[1] V. A. Saveleva, L. Wang, W. Luo, S. Zafeiratos, C. Ulhaq-Bouillet, A. S. Gago, K. A. Friedrich, E. R. Savinova, *J. Phys. Chem. Lett.* **2016**, *7*, 3240.

[2] V. Pfeifer, T. E. Jones, J.-J. Velasco Vélez, M. Greiner, C. Massué, R. Arrigo, D. Teschner, F. Girgsdies, M. Scherzer, J. Allan, M. Hashagen, G. Weinberg, S. Piccinin, M. Haevecker, A. Knop-Gericke, R. Schlögl, *Phys. Chem. Chem. Phys.* **2015**, 1.

[3] H. Y. Hall, M. A. Sherwood, *J Chem Soc, Faraday Trans I* **1984**, *80*, 135.

[4] B. Folkesson, *Acta Chem. Scand.* **1973**, *27*, 287.

[5] R. G. Haverkamp, A. T. Marshall, B. C. C. Cowie, *Surf. Interface Anal.* **2011**, *43*, 847.

[6] M. Peuckert, *Surf. Sci.* **1984**, *144*, 451.

[7] T. Reier, I. Weidinger, P. Hildebrandt, R. Kraehnert, P. Strasser, *ECS Trans.* **2013**, *58*, 39.

[8] J. Augustynski, M. Koudelka, J. Sanchez, B. E. Conway, *J. Electroanal. Chem.* **1984**, *160*, 233.

[9] P. Lettenmeier, R. Wang, R. Abouatallah, S. Helmly, T. Morawietz, R. Hiesgen, S. Kolb, F. Burggraf, J. Kallo, A. S. Gago, K. A. Friedrich, *Electrochim. Acta* **2016**, *210*, 502.
